# Synthesis, Antimicrobial, and Computational Evaluation of Novel Isobutylchalcones as Antimicrobial Agents

**DOI:** 10.1155/2017/6873924

**Published:** 2017-12-26

**Authors:** Afzal Basha Shaik, Rajendra Prasad Yejella, Shahanaaz Shaik

**Affiliations:** ^1^A.U. College of Pharmaceutical Sciences, Andhra University, Visakhapatnam, Andhra Pradesh 53000, India; ^2^Vignan Pharmacy College, Vadlamudi, Andhra Pradesh 522213, India; ^3^Victoria College of Pharmacy, Nallapadu, Andhra Pradesh 522001, India

## Abstract

A series of 25 new chalcones were synthesized by Claisen-Schmidt condensation, well characterized by spectroscopic data, and evaluated for their antibacterial and antifungal activities by serial tube dilution method. Among the compounds tested,** A3 **and** A6 **containing 2,4-dichlorophenyl and 2,4-difluorophenyl moiety, respectively, were found to be the most potent in the series against both bacterial and fungal strains with a MIC value of 16 *µ*g/mL in each case. Further computational evaluation for antimicrobial activity was performed by atom based 3D-QSAR using PHASE™ software in order to have a correlation between the observed activities and predicted activities. The computational studies were in agreement with the* in vitro* antimicrobial results and had identified the most promising chalcones as antimicrobial agents and the responsible structural features for the proposed activity.

## 1. Introduction

The use of antimicrobial agents is critical for the successful treatment of infectious diseases. The existing batteries of antimicrobial agents we have in hand for the treatment of infectious diseases are insufficient to protect us over the long term [1–3]. The primary reason for this situation is inevitable drive of evolution that leads to antimicrobial resistance. At the same time, the nature of new emerging infections is depressing the field of science to predict with accuracy. Resistance to number of antimicrobial agents among a variety of clinically significant species of bacteria is becoming increasingly important global problem [4]. There are various problems arising with the use of antimicrobials such as local tissue irritation, interference with wound healing process, hypersensitivity reactions, systemic toxicity, narrow antimicrobial spectrum, and emergence of resistance. So the increasing clinical importance of drug-resistant microbial pathogens has lent additional urgency in antimicrobial research [5]. Hence there is a compelling need for designing and synthesizing novel drugs of potent, selective, shorter length treatments with less toxic antimicrobial drugs to fight against these lethal infectious diseases [6, 7].

**Figure 1:**
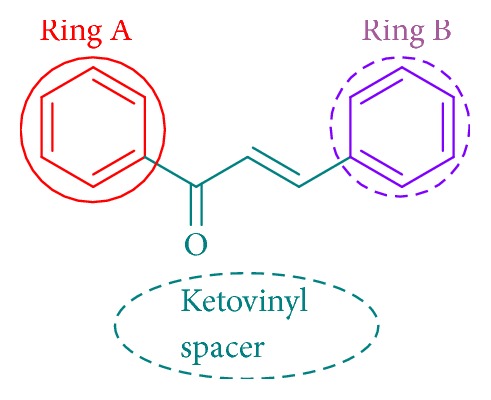
Structure of chalcone.

Chalcones are a group of natural products containing two aryl rings (rings A and B) connected through a three-carbon spacer in the form of ketovinyl group (Figure 1). The spacer is reactive and is responsible for many of the biological activities of these compounds. The various biological activities exhibited by these compounds were reviewed [8–11] and some of them comprise antimicrobial [12–17], antitubercular [18, 19], anticancer [20–23], antioxidant [24, 25], antiprotozoal [26], anthelmintic [27], antimalarial [28], antiulcer [29], analgesic, and anti-inflammatory ones [30–32]. Among the above much of the work was done on the antimicrobial, anticancer, and anti-inflammatory activities and was reviewed [33, 34]. In the recent past, some researchers studied the antimicrobial potentiality of a range of chalcones containing different ring A and B portions [15, 35, 36] but not containing 4-isobutylphenyl moiety. Motivated by these previous studies on antimicrobial properties of chalcones, in the present work we synthesized a series of 4-isobutylacetophenone chalcones with variations in the ring B portion to study the influence of such substituents on antibacterial and antifungal activity of these compounds. Out of the 25 compounds, cytotoxic activity of the twenty compounds** (A1–A20)** was reported as a part of our study on isobutyl chalcones [37]. Herein we have synthesized additional five new compounds** (A21–A25)** in order to frame well-defined structure activity relationships for the proposed antimicrobial activity.

## 2. Materials and Methods

### 2.1. General

All the chemicals used were of analytical grade and purchased from SD Fine and Himedia. 4-Isobutylacetophenone was obtained from Aldrich Chemical Co. Silica gel-G for TLC (Merck) was used as stationary phase and ethyl acetate : hexane (2 : 8) as mobile phase to check the purity of the compounds. UV light (254 nm) and iodine vapours were used to visualize the spots. Melting points were determined in open capillaries, using Boitus melting point apparatus, expressed in °C, and are uncorrected. The IR spectra were recorded using Bruker Vertex 80v spectrometer. ^1^H and ^13^C NMR spectra were recorded on Bruker AMX 400 MHz and chemical shifts are given in units as per million, downfield from tetramethylsilane (TMS) as the internal standard. MS spectra were recorded on Agilent LC-MS spectrometer and elemental analyses were carried out using a Carlo Erba 1108 elemental analyzer for C, H, and N.

### 2.2. Chemistry

#### 2.2.1. Protocol for the Synthesis of Chalcones

**Scheme 1:**
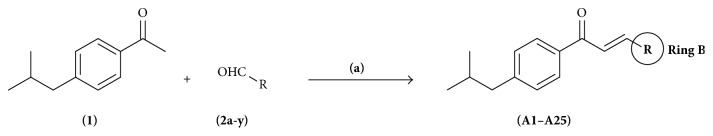
Synthesis of chalcones** (A1–A25)**. Reagents and conditions: (a) ethanol, KOH, and room temperature; (1) 1-(4-isobutylphenyl)ethanone; (2a-y) aromatic or heteroaromatic aldehyde. R = ring B;** A1**: 4′′-chlorophenyl;** A2**: 4′′-methylphenyl;** A3**: 2′′,4′′-dichlorophenyl;** A4**: 2′′-chlorophenyl;** A5**: 4′′-fluorophenyl;** A6**: 2′′,4′′-difluorophenyl;** A7**: 4′′-dimethylaminophenyl;** A8**: 3′′-bromophenyl;** A9**: 4′′-hydroxyphenyl;** A10**: 3′′-hydroxyphenyl;** A11**: 4′′-nitrophenyl;** A12**: 4′′-methoxyphenyl;** A13**: 3′′,4′′-dimethoxyphenyl;** A14**: 3′′,4′′,5′′-trimethoxyphenyl;** A15**: 2′′-pyridinyl;** A16**: 3′′-pyridinyl;** A17**: 4′′-pyridinyl;** A18**: 2′′-pyrrolyl;** A19**: 2′′-thienyl;** A20**: 5′′-bromofuran-2′′-yl;** A21**: 3′′,4′′-methylenedioxyphenyl;** A22**: 9′′-anthracenyl;** A23**: 1′′-phenyl-3′′-methylpyrazole-4′′-yl;** A24**: 3′′-methoxy-4′′-hydroxyphenyl;** A25**: 2′′-chloro-5′′-nitrophenyl.

Equimolar quantities of 4-isobutylacetophenone (0.001 moles) and the appropriate aldehyde (0.001 moles) were dissolved in ethanol (7.5 mL). To this mixture 7.5 mL of 50% aqueous KOH was added dropwise and the reaction mixture was left for 24 h at room temperature. Later, it was acidified with a mixture of hydrochloric acid and water (1 : 1), which resulted in the precipitation of target compounds** (A1–A25)**. The chalcones were then filtered under vacuum, washed with water, dried, and recrystallized from ethanol (Scheme 1) [38, 39].

#### 2.2.2. (E)-1-(4′-Isobutylphenyl)-3-(4′′-chlorophenyl)-2-propen-1-one (A1)

Yield 92%; m.p. 136–138°C; IR (KBr, cm^−1^): 1659 (C=O), 1585 (C=C of Ar), 1505 (CH=CH), 835 (C-Cl), 3050 (Ar C-H), 2833 (Alkyl C-H); ^1^H NMR (400 MHz, CDCl_3_, ppm): *δ* 7.39 (1H, d, *J* = 17 Hz, -CO-CH=), 7.74 (1H, d, *J* = 17 Hz, =CH-Ar), 7.19–7.91 (8H, Ar-H), 0.92 (6H, d, *J* = 8 Hz, -(CH_3_)_2_), 1.75–1.95 (1H, m, -CH-), 2.72 (2H, d, *J* = 8 Hz, -CH_2_-); ^13^C NMR (100 MHz, CDCl_3_, ppm): *δ* 189.77 (C-1), 122.65 (C-2), 142.79 (C-3), 129.41 (C-2′ and C-6′), 129.52 (C-3′ and C-5′), 135.79 (C-1′), 147.53 (C-4′), 133.58 (C-1′′), 138.12 (C-4′′), 128.50 (C-2′′ and C-6′′), 129.22 (C-3′′ and C-5′′), 22.33 (-CH_3_, C of isobutyl group at C-4′′), 30.12 (-CH-, C of isobutyl group at C-4′′), 45.45 (-CH_2_-, C of isobutyl group at C-4′′); MS (*m*/*z*, %): 299.1 (M + 1, 99.16); Anal. Calcd for: C_19_H_19_ClO: C, 76.37; H, 6.41; Found: C, 76.40; H, 6.44.

#### 2.2.3. (E)-1-(4′-Isobutylphenyl)-3-(4′′-methylphenyl)-2-propen-1-one (A2)

Yield 87%; m.p. 128–13°C; IR (KBr, cm^−1^): 1655 (C=O), 1602 (C=C of Ar), 1505 (CH=CH), 3010 (Ar C-H), 2921 (Alkyl C-H); ^1^H NMR (400 MHz, CDCl_3_, ppm): *δ* 2.30 (3H, s, Ar-CH_3_), 7.25 (1H, d, *J* = 17 Hz, -CO-CH=), 7.65 (1H, d, *J* = 17 Hz, =CH-Ar), 6.83–7.82 (8H, Ar-H), 0.89 (6H, d, *J* = 8 Hz, -(CH_3_)_2_), 1.70–1.92 (1H, m, -CH-), 2.65 (2H, d, *J* = 8 Hz, -CH_2_-); ^13^C NMR (100 MHz, CDCl_3_, ppm): *δ* 188.67 (C-1), 121.65 (C-2), 140.97 (C-3), 126.51 (C-2′ and C-6′), 128.22 (C-3′ and C-5′), 132.12 (C-1′), 137.63 (C-4′), 134.89 (C-1′′), 145.12 (C-4′′), 127.09 (C-2′′ and C-6′′), 129.1 (C-3′′ and C-5′′), 22.80 (-CH_3_, C of isobutyl group at C-4′′), 29.12 (-CH-, C of isobutyl group at C-4′′), 45.72 (-CH_2_-, C of isobutyl group at C-4′′), 24.35 (-CH_3_ C at C-4′′); MS (*m*/*z*, %): 279.3 (M + 1, 99.08); Anal. Calcd for: C_20_H_22_O: C, 86.29; H, 7.97; Found: C, 86.32; H, 7.99.

#### 2.2.4. (E)-1-(4′-Isobutylphenyl)-3-(2′′,4′′-dichlorophenyl)-2-propen-1-one (A3)

Yield 85%; m.p. 149–151°C; IR (KBr, cm^−1^): 1655 (C=O), 1581 (C=C of Ar), 1510 (CH=CH), 833 (C-Cl), 3057 (Ar C-H), 2877 (Alkyl C-H); ^1^H NMR (400 MHz, CDCl_3_, ppm): *δ* 7.42 (1H, d, *J* = 17 Hz, -CO-CH=), 7.84 (1H, d, *J* = 17 Hz, =CH-Ar), 7.20–8.20 (7H, Ar-H), 1.11 (6H, d, *J* = 8 Hz, -(CH_3_)_2_), 1.99–2.13 (1H, m, -CH-), 2.73 (2H, d, *J* = 8 Hz, -CH_2_-); ^13^C NMR (100 MHz, CDCl_3_, ppm): *δ* 190.11 (C-1), 121.78 (C-2), 141.21 (C-3), 130.03 (C-2′ and C-6′), 130.35 (C-3′ and C-5′), 134.25 (C-1′), 147.63 (C-4′), 132.71 (C-1′′), 135.27 (C-4′′), 132.69 (C-2′′), 129.23 (C-6′′), 130.79 (C-3′′), 126.72 (C-5′′), 23.11 (-CH_3_, C of isobutyl group at C-4′′), 29.31 (-CH-, C of isobutyl group at C-4′′), 45.91 (-CH_2_-, C of isobutyl group at C-4′′); MS (*m*/*z*, %): 334.1 (M + 1, 99.00); Anal. Calcd for: C_19_H_18_Cl_2_O: C, 68.48; H, 5.44; Found: C, 68.53; H, 5.49.

#### 2.2.5. (E)-1-(4′-Isobutylphenyl)-3-(2′′-chlorophenyl)-2-propen-1-one (A4)

Yield 65%; m.p. 140–142°C; IR (KBr, cm^−1^): 1652 (C=O), 1583 (C=C of Ar), 1502 (CH=CH), 833 (C-Cl), 3120 (Ar C-H), 2920 (Alkyl C-H); ^1^H NMR (400 MHz, CDCl_3_, ppm): *δ* 7.31 (1H, d, *J* = 17 Hz, -CO-CH=), 7.74 (1H, d, *J* = 17 Hz, =CH-Ar), 6.87–7.91 (8H, Ar-H), 1.02 (6H, d, *J* = 8 Hz, -(CH_3_)_2_), 2.22–2.44 (1H, m, -CH-), 2.66 (2H, d, *J* = 8 Hz, -CH_2_-); ^13^C NMR (100 MHz, CDCl_3_, ppm): *δ* 189.44 (C-1), 121.42 (C-2), 145.15 (C-3), 128.5 (C-2′ and C-6′), 129.5 (C-3′ and C-5′), 133.22 (C-1′), 146.71 (C-4′), 133.91 (C-1′′), 129.55 (C-4′′), 128.85 (C-2′′), 130.64 (C-6′′), 128.8 (C-3′′), 126.2 (C-5′′), 22.21 (-CH_3_, C of isobutyl group at C-4′′), 29.11 (-CH-, C of isobutyl group at C-4′′), 45.71 (-CH_2_-, C of isobutyl group at C-4′′); MS (*m*/*z*, %): 299.8 (M + 1, 99.22); Anal. Calcd for: C_19_H_19_ClO: C, 76.37; H, 6.41; Found: C, 76.42; H, 6.50.

#### 2.2.6. (E)-1-(4′-Isobutylphenyl)-3-(4′′-fluorophenyl)-2-propen-1-one (A5)

Yield 85%; m.p. 142–144°C; IR (KBr, cm^−1^): 1664 (C=O), 1580 (C=C of Ar), 1524 (CH=CH), 928 (C-F), 3127 (Ar C-H), 2954 (Alkyl C-H); ^1^H NMR (400 MHz, CDCl_3_, ppm): *δ* 7.57 (1H, d, *J* = 17 Hz, -CO-CH=), 7.87 (1H, d, *J* = 17 Hz, =CH-Ar), 7.33–8.12 (8H, Ar-H), 1.00 (6H, d, *J* = 8 Hz, -(CH_3_)_2_), 1.80–2.04 (1H, m, -CH-), 2.75 (2H, d, *J* = 8 Hz, -CH_2_-); ^13^C NMR (100 MHz, CDCl_3_, ppm): *δ* 190.21 (C-1), 124.52 (C-2), 145.29 (C-3), 129.44 (C-2′ and C-6′), 129.67 (C-3′ and C-5′), 135.92 (C-1′), 144.76 (C-4′), 131.8 (C-1′′), 163.12 (C-4′′), 129.11 (C-2′′ and C-6′′), 118.98 (C-3′′ and C-5′′), 22.82 (-CH_3_, C of isobutyl group at C-4′′), 29.57 (-CH-, C of isobutyl group at C-4′′), 45.91 (-CH_2_-, C of isobutyl group at C-4′′); MS (*m*/*z*, %): 283.3 (M + 1, 99.08); Anal. Calcd for: C_19_H_19_FO: C, 80.82; H, 6.78; Found: C, 80.86; H, 6.85.

#### 2.2.7. (E)-1-(4′-Isobutylphenyl)-3-(2′′,4′′-difluorophenyl)-2-propen-1-one (A6)

Yield 79%; m.p. 163–165°C; IR (KBr, cm^−1^): 1655 (C=O), 1581 (C=C of Ar), 1510 (CH=CH), 925 (C-F), 926 (C-F), 3040 (Ar C-H), 2933 (Alkyl C-H); ^1^H NMR (400 MHz, CDCl_3_, ppm): *δ* 7.49 (1H, d, *J* = 17 Hz, -CO-CH=), 7.99 (1H, d, *J* = 17 Hz, =CH-Ar), 7.11–8.20 (7H, Ar-H), 1.19 (6H, d, *J* = 8 Hz, -(CH_3_)_2_), 2.10–2.41 (1H, m, -CH-), 2.91 (2H, d, *J* = 8 Hz, -CH_2_-); ^13^C NMR (100 MHz, CDCl_3_, ppm): *δ* 190.23 (C-1), 122.1 (C-2), 146.2 (C-3), 134.8 (C-2′ and C-6′), 129.52 (C-3′ and C-5′), 134.55 (C-1′), 147.29 (C-4′), 134.11 (C-1′′), 165.42 (C-4′′), 159.51 (C-2′′), 129.6 (C-6′′), 109.29 (C-3′′), 112.0 (C-5′′), 23.21 (-CH_3_, C of isobutyl group at C-4′′), 30.12 (-CH-, C of isobutyl group at C-4′′), 45.81 (-CH_2_-, C of isobutyl group at C-4′′); MS (*m*/*z*, %): 301.3 (M + 1, 99.01); Anal. Calcd for: C_19_H_19_FO: C, 75.98; H, 6.04; Found: C, 76.03; H, 6.06.

#### 2.2.8. (E)-1-(4′-Isobutylphenyl)-3-(4′′-dimethylaminophenyl)-2-propen-1-one (A7)

Yield 82%; m.p. 138–140°C; IR (KBr, cm^−1^): 1650 (C=O), 1586 (C=C of Ar), 1505 (CH=CH), 1178 (-N(CH_3_)_2_), 3198 (Ar C-H), 2940 (Alkyl C-H); ^1^H NMR (100 MHz, CDCl_3_, ppm): *δ* 3.10 (6H, s, -N(CH_3_)_2_), 7.29 (1H, d, *J* = 17 Hz, -CO-CH=), 7.75 (1H, d, *J* = 17 Hz, =CH-Ar), 6.64–8.10 (8H, Ar-H), 0.98 (6H, d, *J* = 8 Hz, -(CH_3_)_2_), 2.19–2.33 (1H, m, -CH-), 2.58 (2H, d, *J* = 8 Hz, -CH_2_-); ^13^C NMR (100 MHz, CDCl_3_, ppm): *δ* 186.61 (C-1), 120.10 (C-2), 144.65 (C-3), 130.11 (C-2′ and C-6′), 128.45 (C-3′ and C-5′), 135.67 (C-1′), 146.81 (C-4′), 133.31 (C-1′′) 167.22 (C-4′′), 159.51 (C-2′′), 129.61 (C-6′′), 109.29 (C-3′′), 111.03 (C-5′′), 23.10 (-CH_3_, C of isobutyl group at C-4′′), 30.33 (-CH-, C of isobutyl group at C-4′′), 45.99 (-CH_2_-, C of isobutyl group at C-4′′), 40.33 (-N(CH_3_)_2_,); MS (*m*/*z*, %): 308.4 (M + 1, 99.09); Anal. Calcd for: C_21_H_25_NO: C, 82.04; H, 8.20; N, 4.56; Found: C, 82.09; H, 8.23; N, 4.59.

#### 2.2.9. (E)-1-(4′-Isobutylphenyl)-3-(3′′-bromophenyl)-2-propen-1-one (A8)

Yield 80%; m.p. 107–109°C; IR (KBr, cm^−1^): 1650 (C=O), 1605 (C=C of Ar), 1502 (CH=CH), 969 (C-Br), 3155 (Ar C-H), 2836 (Alkyl C-H); ^1^H NMR (400 MHz, CDCl_3_, ppm): *δ* 7.36 (1H, d, *J* = 17 Hz, -CO-CH=), 7.79 (1H, d, *J* = 17 Hz, =CH-Ar), 7.19–8.09 (8H, Ar-H), 1.01 (6H, d, *J* = 8 Hz, -(CH_3_)_2_), 2.02–2.20 (1H, m, -CH-), 2.58 (2H, d, *J* = 8 Hz, -CH_2_-); ^13^C NMR (100 MHz, CDCl_3_, ppm): *δ* 189.37 (C-1), 123.09 (C-2), 146.27 (C-3), 131.21 (C-2′ and C-6′), 129.27 (C-3′ and C-5′), 136.13 (C-1′), 146.28 (C-4′), 137.42 (C-1′′), 131.12 (C-4′′ and C-5′′), 130.54 (C-2′′), 124.43 (C-6′′), 127.67 (C-3′′), 22.92 (-CH_3_, C of isobutyl group at C-4′′), 30.12 (-CH-, C of isobutyl group at C-4′′), 45.61 (-CH_2_-, C of isobutyl group at C-4′′); MS (*m*/*z*, %): 343.2 (M + 1, 99.21); Anal. Calcd for: C_19_H_19_BrO: C, 66.48; H, 5.58; Found: C, 66.53; H, 5.63.

#### 2.2.10. (E)-1-(4′-Isobutylphenyl)-3-(4′′-hydroxyphenyl)-2-propen-1-one (A9)

Yield 73%; m.p. 156–158°C; IR (KBr, cm^−1^): 3460 (O-H), 1648 (C=O), 1606 (C=C of Ar), 1505 (CH=CH), 3060 (Ar C-H), 2852 (Alkyl C-H); ^1^H NMR (100 MHz, CDCl_3_, ppm): *δ* 4.92 (1H, Ar-OH), 7.29 (1H, d, *J* = 17 Hz, -CO-CH=), 7.80 (1H, d, *J* = 17 Hz, =CH-Ar), 7.61–8.02 (8H, Ar-H), 1.02 (6H, d, *J* = 8 Hz, -(CH_3_)_2_), 1.92–2.01 (1H, m, -CH-), 2.40 (2H, d, *J* = 8 Hz, -CH_2_-); ^13^C NMR (100 MHz, CDCl_3_, ppm): *δ* 188.52 (C-1), 122.87 (C-2), 148.32 (C-3), 131.26 (C-2′ and C-6′), 128.91 (C-3′ and C-5′), 134.99 (C-1′), 145.56 (C-4′), 133.54 (C-1′′), 159.35 (C-4′′), 129.11 (C-2′′ and C-6′′), 120.21 (C-3′′ and C-5′′), 23.45 (-CH_3_, C of isobutyl group at C-4′), 29.92 (-CH-, C of isobutyl group at C-4′), 44.99 (-CH_2_-, C of isobutyl group at C-4′); MS (*m*/*z*, %): 281.3 (M + 1, 99.16); Anal. Calcd for: C_19_H_19_O_2_: C, 81.40; H, 7.19; Found: C, 81.45; H, 7.24.

#### 2.2.11. (E)-1-(4′-Isobutylphenyl)-3-(3′′-hydroxyphenyl)-2-propen-1-one (A10)

Yield 65%; m.p. 152–154°C; IR (KBr, cm^−1^): 3520 (O-H), 1648 (C=O), 1612 (C=C of Ar), 1505 (CH=CH), 3111 (Ar C-H), 2928 (Alkyl C-H); ^1^H NMR (400 MHz, CDCl_3_, ppm): *δ* 4.80 (1H, Ar-OH), 7.26 (1H, d, *J* = 17 Hz, -CO-CH=), 7.71 (1H, d, *J* = 17 Hz, =CH-Ar), 6.85–8.00 (8H, Ar-H), 1.01 (6H, d, *J* = 8 Hz, -(CH_3_)_2_), 2.19–2.31 (1H, m, -CH-), 2.45 (2H, d, *J* = 8 Hz, -CH_2_-); ^13^C NMR (100 MHz, CDCl_3_, ppm): *δ* 188.96 (C-1), 123.13 (C-2), 148.77 (C-3), 132.11 (C-2′ and C-6′), 129.56 (C-3′ and C-5′), 135.43 (C-1′), 146.12 (C-4′), 136.62 (C-1′′), 115.21 (C-2′′), 159.43 (C-3′′), 118.46 (C-4′′), 130.13 (C-5′′), 120.14 (C-6′′), 22.67 (-CH_3_, C of isobutyl group at C-4′), 29.39 (-CH-, C of isobutyl group at C-4′), 44.17 (-CH_2_-, C of isobutyl group at C-4′); MS (*m*/*z*, %): 280.3 (M + 1, 99.10); Anal. Calcd for: C_19_H_19_O_2_: C, 81.40; H, 7.19; Found: C, 81.45; H, 7.24.

#### 2.2.12. (E)-1-(4′-Isobutylphenyl)-3-(4′′-nitrophenyl)-2-propen-1-one (A11)

Yield 95%; m.p. 190–192°C; IR (KBr, cm^−1^): 1652 (C=O), 1610 (C=C of Ar), 1502 (CH=CH), 1541 (N=O, asymmetric), 1346 (N=O, symmetric), 3092 (Ar C-H), 2951 (Alkyl C-H). ^1^H NMR (400 MHz, CDCl_3_, *δ*): 7.35 (1H, d, *J* = 17 Hz, -CO-CH=), 7.84 (1H, d, *J* = 17 Hz, =CH-Ar), 7.05–7.95 (8H, Ar-H), 0.91 (6H, d, *J* = 8 Hz, -(CH_3_)_2_), 2.10–2.13 (1H, m, -CH-), 2.33 (2H, d, *J* = 8 Hz, -CH_2_-); ^13^C NMR (100 MHz, CDCl_3_): *δ* 190.22 (C-1), 123.68 (C-2), 147.56 (C-3), 133.13 (C-2′ and C-6′), 131.58 (C-3′ and C-5′), 137.02 (C-1′), 148.31 (C-4′), 142.41 (C-1′′), 152.32 (C-4′′), 128.23 (C-2′′ and C-6′′), 122.11 (C-3′′ and C-5′′), 22.18 (-CH_3_, C of isobutyl group at C-4′), 28.98 (-CH-, C of isobutyl group at C-4′) and 43.87 (-CH_2_-, C of isobutyl group at C-4′); MS (*m*/*z*, %): 310.3 (M + 1, 99.38); Anal. Calcd for: C_19_H_19_NO_3_: C, 73.77; H, 6.19; N, 4.53; Found: C, 73.80; H, 6.24; N, 4.59.

#### 2.2.13. (E)-1-(4′-Isobutylphenyl)-3-(4′′-methoxyphenyl)-2-propen-1-one (A12)

Yield 79%; m.p. 149–151°C; IR (KBr, cm^−1^): 1655 (C=O), 1605 (C=C of Ar), 1508 (CH=CH), 1125 (-OCH_3_), 3054 (Ar C-H), 2956 (Alkyl C-H); ^1^H NMR (400 MHz, CDCl_3_, ppm): *δ* 3.90 (3H, s, Ar-OCH_3_), 7.19 (1H, d, *J* = 17 Hz, -CO-CH=), 7.74 (1H, d, *J* = 17 Hz, =CH-Ar), 6.71–8.08 (8H, Ar-H), 0.80 (6H, d, *J* = 8 Hz, -(CH_3_)_2_), 1.62–1.84 (1H, m, -CH-), 2.09 (2H, d, *J* = 8 Hz, -CH_2_-); ^13^C NMR (100 MHz, CDCl_3_, ppm): *δ* 189.05 (C-1), 121.12 (C-2), 142.97 (C-3), 127.85 (C-2′ and C-6′), 129.51 (C-3′ and C-5′), 133.53 (C-1′), 143.32 (C-4′), 134.89 (C-1′′) 145.12 (C-4′′), 127.09 (C-2′′ and C-6′′), 115.51 (C-3′′ and C-5′′), 22.31 (-CH_3_, C of isobutyl group at C-4′′), 28.91 (-CH-, C of isobutyl group at C-4′′), 44.91 (-CH_2_-, C of isobutyl group at C-4′′), 55.99 (-OCH_3_ C at C-4′′); MS (*m*/*z*, %): 295.1 (M + 1, 99.28); Anal. Calcd for: C_20_H_22_O_2_: C, 81.60; H, 7.53; Found: C, 81.65; H, 7.57.

#### 2.2.14. (E)-1-(4′-Isobutylphenyl)-3-(3′′,4′′-dimethoxyphenyl)-2-propen-1-one (A13)

Yield 66%; m.p. 146–148°C; IR (KBr, cm^−1^): 1655 (C=O), 1605 (C=C of Ar), 1500 (CH=CH), 1130 (-OCH_3_), 3066 (Ar C-H), 2839 (Alkyl C-H); ^1^H NMR (400 MHz, CDCl_3_, ppm): *δ* 3.95 (6H, s, 2x Ar-OCH_3_), 7.21 (1H, d, *J* = 17 Hz, -CO-CH=), 7.80 (1H, d, *J* = 17 Hz, =CH-Ar), 6.91–8.12 (6H, Ar-H), 1.00 (6H, d, *J* = 8 Hz, -(CH_3_)_2_), 2.21–2.42 (1H, m, -CH-), 2.55 (2H, d, *J* = 8 Hz, -CH_2_-); ^13^C NMR (100 MHz, CDCl_3_, ppm): *δ* 188.55 (C-1), 121.01 (C-2), 142.31 (C-3), 129.61 (C-2′ and C-6′), 130.74 (C-3′ and C-5′), 134.20 (C-1′), 144.53 (C-4′), 128.29 (C-1′′) 112.22 (C-2′′), 149.90 (C-3′′ and C-4′′), 115.51 (C-5′′), 119.84 (C-6′′), 21.71 (-CH_3_, C of isobutyl group at C-4′′), 28.58 (-CH-, C of isobutyl group at C-4′′), 43.82 (-CH_2_-, C of isobutyl group at C-4′′), 56.71 (-OCH_3_ C at C-3′′ and C-4′′); 325.4 (M + 1, 99.42); Anal. Calcd for: C_20_H_22_O_2_: C, 77.75; H, 7.46; Found: C, 77.77; H, 7.47.

#### 2.2.15. (E)-1-(4′-Isobutylphenyl)-3-(3′′,4′′,5′′-trimethoxyphenyl)-2-propen-1-one (A14)

Yield 70%; m.p. 180–182°C; IR (KBr, cm^−1^): 1652 (C=O), 1585 (C=C of Ar), 1462 (CH=CH), 1127 (-OCH_3_), 3110 (Ar C-H), 2853 (Alkyl C-H); ^1^H NMR (400 MHz, CDCl_3_, ppm): *δ* 3.90 (3H, s, Ar-OCH_3_), 3.92 (6H, s, 2x Ar-OCH_3_), 7.22 (1H, d, *J* = 17 Hz, -CO-CH=), 7.53 (1H, d, *J* = 17 Hz, =CH-Ar), 6.85–8.07 (6H, Ar-H), 1.08 (6H, d, *J* = 8 Hz, -(CH_3_)_2_), 2.29–2.45 (1H, m, -CH-), 2.65 (2H, d, *J* = 8 Hz, -CH_2_-); ^13^C NMR (100 MHz, CDCl_3_, ppm): *δ* 188.11 (C-1), 121.33 (C-2), 141.86 (C-3), 128.11 (C-2′ and C-6′), 130.99 (C-3′ and C-5′), 134.73 (C-1′), 146.24 (C-4′), 129.93 (C-1′′), 102.91 (C-2′′ and C-6′′), 151.04 (C-3′′ and C-5′′), 139.29 (C-4′′), 21.91 (-CH_3_, C of isobutyl group at C-4′′), 29.34 (-CH-, C of isobutyl group at C-4′′), 45.56 (-CH_2_-, C of isobutyl group at C-4′′), 56.71 (-OCH_3_ C at C-3′′, C-4′′ and C-5′′); MS (*m*/*z*, %): 355.4 (M + 1, 99.06); Anal. Calcd for: C_20_H_22_O_2_: C, 74.55; H, 7.39; Found: C, 74.56; H, 7.43.

#### 2.2.16. (E)-1-(4′-Isobutylphenyl)-3-(2′′-pyridinyl)-2-propen-1-one (A15)

Yield 76%; m.p. 132–134°C; IR (KBr, cm^−1^): 1651 (C=O), 1581 (C=N), 1604 (C=C of Ar), 1505 (CH=CH), 1368 (C-N), 3006 (Ar C-H), 2799 (Alkyl C-H); ^1^H NMR (400 MHz, CDCl_3_, ppm): *δ* 7.15 (1H, d, *J* = 17 Hz, -CO-CH=), 7.51 (1H, d, *J* = 17 Hz, =CH-Ar), 6.32–8.41 (8H, Ar-H), 1.91 (6H, d, *J* = 8 Hz, -(CH_3_)_2_), 1.89–2.09 (1H, m, -CH-), 2.33 (2H, d, *J* = 8 Hz, -CH_2_-); ^13^C NMR (100 MHz, CDCl_3_, ppm): *δ* 189.70 (C-1), 127.73 (C-2), 140.32 (C-3), 128.39 (C-2′ and C-6′), 129.05 (C-3′ and C-5′), 134.94 (C-1′), 147.11 (C-4′), 155.75 (C-2′′), 122.02 (C-3′′), 137.39 (C-4′′), 123.09 (C-5′′), 149.16 (C-5′′), 22.53 (-CH_3_, C of isobutyl group at C-4′′), 29.88 (-CH-, C of isobutyl group at C-4′′), 45.99 (-CH_2_-, C of isobutyl group at C-4′′); MS (*m*/*z*, %): 265.3 (M + 1, 99.32); Anal. Calcd for: C_18_H_19_NO: C, 81.47; H, 7.22; N, 5.28; Found: C, 81.51; H, 7.25; N, 5.33.

#### 2.2.17. (E)-1-(4′-Isobutylphenyl)-3-(3′′-pyridinyl)-2-propen-1-one (A16)

Yield 86%; m.p. 143–145°C; IR (KBr, cm^−1^): 1645 (C=O), 1590 (C=N), 1603 (C=C of Ar), 1502 (CH=CH), 1370 (C-N), 3098 (Ar C-H), 2937 (Alkyl C-H); ^1^H NMR (400 MHz, CDCl_3_, ppm): *δ* 7.17 (1H, d, *J* = 17 Hz, -CO-CH=), 7.55 (1H, d, *J* = 17 Hz, =CH-Ar), 6.23–8.15 (8H, Ar-H), 0.99 (6H, d, *J* = 8 Hz, -(CH_3_)_2_), 1.90–2.13 (1H, m, -CH-), 2.59 (2H, d, *J* = 8 Hz, -CH_2_-); ^13^C NMR (100 MHz, CDCl_3_, ppm): *δ* 188.20 (C-1), 127.31 (C-2), 143.32 (C-3), 127.91 (C-2′ and C-6′), 129.59 (C-3′ and C-5′), 134.77 (C-1′), 146.52 (C-4′), 151.25 (C-2′′), 132.26 (C-3′′), 133.53 (C-4′′), 123.85 (C-5′′), 149.99 (C-5′′), 22.11 (-CH_3_, C of isobutyl group at C-4′′), 29.59 (-CH-, C of isobutyl group at C-4′′), 45.12 (-CH_2_-, C of isobutyl group at C-4′′); MS (*m*/*z*, %): 265.3 (M + 1, 99.18); Anal. Calcd for: C_18_H_19_NO: C, 81.47; H, 7.22; N, 5.28; Found: C, 81.51; H, 7.25; N, 5.33.

#### 2.2.18. (E)-1-(4′-Isobutylphenyl)-3-(4′′-pyridinyl)-2-propen-1-one (A17)

Yield 89%; m.p. 165–167°C; IR (KBr, cm^−1^): 1650 (C=O), 1581 (C=N), 1605 (C=C of Ar), 1505 (CH=CH), 1373 (C-N), 3101 (Ar C-H), 2811 (Alkyl C-H); ^1^H NMR (400 MHz, CDCl_3_, ppm): *δ* 7.26 (1H, d, *J* = 17 Hz, -CO-CH=), 7.61 (1H, d, *J* = 17 Hz, =CH-Ar), 6.21–8.59 (8H, Ar-H), 0.93 (6H, d, *J* = 8 Hz, -(CH_3_)_2_), 2.12–2.17 (1H, m, -CH-), 2.62 (2H, d, *J* = 8 Hz, -CH_2_-); ^13^C NMR (100 MHz, CDCl_3_, ppm): *δ* 188.59 (C-1), 127.77 (C-2), 143.91 (C-3), 128.26 (C-2′ and C-6′), 128.88 (C-3′ and C-5′), 134.96 (C-1′), 146.97 (C-4′), 149.35 (C-2′′), 121.75 (C-3′′), 144.31 (C-4′′), 120.92 (C-5′′), 149.97 (C-5′′), 22.11 (-CH_3_, C of isobutyl group at C-4′′), 29.59 (-CH-, C of isobutyl group at C-4′′), 45.12 (-CH_2_-, C of isobutyl group at C-4′′); MS (*m*/*z*, %): 265.3 (M + 1, 99.20); Anal. Calcd for: C_18_H_19_NO: C, 81.47; H, 7.22; N, 5.28; Found: C, 81.51; H, 7.25; N, 5.33.

#### 2.2.19. (E)-1-(4′-Isobutylphenyl)-3-(2′′-pyrrolyl)-2-propen-1-one (A18)

Yield 82%; m.p. 189–191°C; IR (KBr, cm^−1^): 1652 (C=O), 1588 (C=N), 1605 (C=C of Ar), 1506 (CH=CH), 1375 (C-N), 3121 (Ar C-H), 2935 (Alkyl C-H); ^1^H NMR (400 MHz, CDCl_3_, ppm): *δ* 5.10 (1H, s, -NH), 7.24 (1H, d, *J* = 17 Hz, -CO-CH=), 7.60 (1H, d, *J* = 17 Hz, =CH-Ar), 6.94–7.72 (7H, Ar-H), 0.95 (6H, d, *J* = 8 Hz, -(CH_3_)_2_), 1.85–2.07 (1H, m, -CH-), 2.55 (2H, d, *J* = 8 Hz, -CH_2_-); ^13^C NMR (100 MHz, CDCl_3_, ppm): *δ* 187.51 (C-1), 126.23 (C-2), 132.74 (C-3), 129.17 (C-2′ and C-6′), 129.88 (C-3′ and C-5′), 134.61 (C-1′), 146.97 (C-4′), 129.51 (C-2′′), 112.56 (C-3′′), 108.26 (C-4′′), 119.39 (C-5′′), 23.12 (-CH_3_, C of isobutyl group at C-4′′), 30.63 (-CH-, C of isobutyl group at C-4′′), 47.99 (-CH_2_-, C of isobutyl group at C-4′′); MS (*m*/*z*, %): 299.1 (M + 1, 99.16); Anal. Calcd for: C_17_H_19_NO: C, 80.60; H, 7.56; N, 5.53; Found: C, 80.64; H, 7.59; N, 5.54.

#### 2.2.20. (E)-1-(4′-Isobutylphenyl)-3-(2′′-thienyl)-2-propen-1-one (A19)

Yield 86%; m.p. 179–181°C; IR (KBr, cm^−1^): 1655 (C=O), 1610 (C=C of Ar), 1505 (CH=CH), 624 (C-S), 3119 (Ar C-H), 2954 (Alkyl C-H); ^1^H NMR (400 MHz, CDCl_3_, ppm): *δ* 7.34 (1H, d, *J* = 17 Hz, -CO-CH=), 7.82 (1H, d, *J* = 17 Hz, =CH-Ar), 6.85–8.30 (7H, Ar-H), 0.85 (6H, d, *J* = 8 Hz, -(CH_3_)_2_), 1.71–2.09 (1H, m, -CH-), 2.99 (2H, d, *J* = 8 Hz, -CH_2_-); ^13^C NMR (100 MHz, CDCl_3_, ppm): *δ* 189.98 (C-1), 127.95 (C-2), 134.26 (C-3), 130.76 (C-2′ and C-6′), 129.76 (C-3′ and C-5′), 135.84 (C-1′), 147.38 (C-4′), 138.85 (C-2′′), 128.21 (C-3′′), 129.15 (C-4′′), 130.19 (C-5′′), 22.91 (-CH_3_, C of isobutyl group at C-4′′), 30.11 (-CH-, C of isobutyl group at C-4′′), 47.12 (-CH_2_-, C of isobutyl group at C-4′′); MS (*m*/*z*, %): 254.3 (M + 1, 99.04); Anal. Calcd for: C_17_H_18_SO: C, 75.51; H, 6.71; Found: C, 75.55; H, 6.73.

#### 2.2.21. (E)-1-(4′-Isobutylphenyl)-3-(5′′-bromofuran-2′′-yl)-2-propen-1-one (A20)

Yield 85%; m.p. 149–151°C; IR (KBr, cm^−1^): 1652 (C=O), 1585 (C=C of Ar), 1503 (CH=CH), 2959 (Ar C-H), 2713 (Alkyl C-H); ^1^H NMR (400 MHz, CDCl_3_, ppm): *δ* 7.19 (1H, d, *J* = 17 Hz, -CO-CH=), 7.79 (1H, d, *J* = 17 Hz, =CH-Ar), 6.89–7.85 (7H, Ar-H), 1.01 (6H, d, *J* = 8 Hz, -(CH_3_)_2_), 2.05–2.22 (1H, m, -CH-), 2.62 (2H, d, *J* = 8 Hz, -CH_2_-); ^13^C NMR (100 MHz, CDCl_3_, ppm): *δ* 189.88 (C-1), 127.58 (C-2), 131.43 (C-3), 130.21 (C-2′ and C-6′), 128.95 (C-3′ and C-5′), 135.13 (C-1′), 146.88 (C-4′), 154.81 (C-2′′), 114.38 (C-3′′), 114.67 (C-4′′), 123.12 (C-5′′), 22.65 (-CH_3_, C of isobutyl group at C-4′′), 29.81 (-CH-, C of isobutyl group at C-4′′), 46.66 (-CH_2_-, C of isobutyl group at C-4′′); MS (*m*/*z*, %): 334.2 (M + 1, 99.42); Anal. Calcd for: C_17_H_17_BrO_2_: C, 61.28; H, 5.14; Found: C, 61.33; H, 5.17.

#### 2.2.22. (E)-1-(4′-Isobutylphenyl)-3-(3′′,4′′-methylenedioxyphenyl)-2-propen-1-one (A21)

Yield 78%; m.p. 102–104°C; IR (KBr, cm^−1^): 1643 (C=O), 1574 (C=C of Ar), 1500 (CH=CH), 1240 (O-CH_2_-O), 3122 (Ar C-H), 2963 (Alkyl C-H); ^1^H NMR (400 MHz, CDCl_3_, ppm): *δ* 5.92 (2H, s, -O-CH_2_O-), 7.25 (1H, d, *J* = 17 Hz, -CO-CH=), 7.77 (1H, d, *J* = 17 Hz, =CH-Ar), 6.84–8.09 (7H, Ar-H), 1.10 (6H, d, *J* = 8 Hz, -(CH_3_)_2_), 2.33–2.51 (1H, m, -CH-), 2.66 (2H, d, *J* = 8 Hz, -CH_2_-); ^13^C NMR (100 MHz, CDCl_3_, ppm): *δ* 188.78 (C-1), 122.76 (C-2), 143.23 (C-3), 129.28 (C-2′ and C-6′), 129.91 (C-3′ and C-5′), 134.43 (C-1′), 147.82 (C-4′), 154.81 (C-2′′), 115.67 (C-3′′), 116.76 (C-4′′), 122.21 (C-5′′), 22.63 (-CH_3_, C of isobutyl group at C-4′′), 29.43 (-CH-, C of isobutyl group at C-4′′), 45.78 (-CH_2_-, C of isobutyl group at C-4′′), 101.25 (-CH_2_-, C of methylenedioxy group); MS (*m*/*z*, %): 309.2 (M + 1, 99.02); Anal. Calcd for: C_20_H_20_O_3_: C, 77.90; H, 6.54; Found: C, 77.94; H, 6.56.

#### 2.2.23. (E)-1-(4′-Isobutylphenyl)-3-(4′′-anthracenyl)-2-propen-1-one (A22)

Yield 66%; m.p. 146–148°C; IR (KBr, cm^−1^): 1658 (C=O), 1605 (C=C of Ar), 1503 (CH=CH), 3011 (Ar C-H), 2883 (Alkyl C-H); ^1^H NMR (400 MHz, CDCl_3_, ppm): *δ* 7.20 (1H, d, *J* = 17 Hz, -CO-CH=), 7.92 (1H, d, *J* = 17 Hz, =CH-Ar), 6.90–9.40 (13H, Ar-H), 0.80 (6H, d, *J* = 8 Hz, -(CH3)2), 1.72–1.92 (1H, m, -CH-), 2.46 (2H, d, *J* = 8 Hz, -CH2-); ^13^C NMR (100 MHz, CDCl_3_, ppm): *δ* 188.55 (C-1), 121.01 (C-2), 142.31 (C-3), 129.61 (C-2′ and C-6′), 130.74 (C-3′ and C-5′), 134.20 (C-1′), 144.53 (C-4′), 121.62–133.12 (14 Cs of anthracenyl ring), 21.71 (-CH_3_, C of isobutyl group at C-4′), 28.58 (-CH-, C of isobutyl group at C-4′′), 43.82 (-CH_2_-, C of isobutyl group at C-4′′), 56.71 (-OCH_3_ C at C-3′′ and C-4′′); MS (*m*/*z*, %): 265.4 (M + 1, 98.8); Anal. Calcd for: C_20_H_22_O_2_: C, 77.75; H, 7.46; Found: C, 77.77; H, 7.47.

#### 2.2.24. (E)-1-(4′-Isobutylphenyl)-3-(1′′-phenyl-3′′methylpyrazole-4′′-yl)-2-propen-1-one (A23)

Yield 60%; m.p. 139–141°C; IR (KBr, cm^−1^): 1663 (C=O), 1610 (C=N), 1588 (C=C of Ar), 1510 (CH=CH), 1391 (C-N), 3105 (Ar C-H), 2732 (Alkyl C-H); ^1^H NMR (400 MHz, CDCl_3_, ppm): *δ* 2.15 (3H, s, -CH3) 7.24 (1H, d, *J* = 17 Hz, -CO-CH=), 7.82 (1H, d, *J* = 17 Hz, =CH-Ar), 6.85–8.11 (10H, Ar-H), 0.85 (6H, d, *J* = 8 Hz, -(CH3)2), 1.75–1.99 (1H, m, -CH-), 2.46 (2H, d, *J* = 8 Hz, -CH2-); ^13^C NMR (100 MHz, CDCl_3_, ppm): *δ* 186.21 (C-1), 122.45 (C-2), 140.81 (C-3), 129.61 (C-2′ and C-6′), 131.07 (C-3′ and C-5′), 134.21 (C-1′), 145.46 (C-4′), 149.22 (C-3′′ of pyrazole ring), 114.64 (C-4′′ of pyrazole ring), 131.71 (C-5′′ of pyrazole ring), 119–130 (6 Carbons of N-1 phenyl of pyrazole), 22.34 (-CH_3_, C of isobutyl group at C-4′′), 29.67 (-CH-, C of isobutyl group at C-4′′), 46.11 (-CH_2_-, C of isobutyl group at C-4′′); MS (*m*/*z*, %): 345.4 (M + 1, 98.18); Anal. Calcd for: C_23_H_24_N_2_O: C, 80.20; H, 7.02; N, 8.13; Found: C, 80.24; H, 7.04; N, 8.14.

#### 2.2.25. (E)-1-(4′-Isobutylphenyl)-3-(3′′-methoxy-4′′-hydroxyphenyl)-2-propen-1-one (A24)

Yield 71%; m.p. 126–128°C; IR (KBr, cm^−1^): 3450 (O-H), 1648 (C=O), 1606 (C=C of Ar), 1510 (CH=CH), 1225 (-OCH_3_), 3001 (Ar C-H), 2833 (Alkyl C-H); ^1^H NMR (400 MHz, CDCl_3_, ppm): *δ* 3.65 (3H, s, -OCH3), 5.20 (1H, s, Ar-OH), 7.29 (1H, d, *J* = 17 Hz, -CO-CH=), 7.80 (1H, d, *J* = 17 Hz, =CH-Ar), 6.91–8.09 (7H, Ar-H), 1.01 (6H, d, *J* = 8 Hz, -(CH3)_2_), 2.22–2.44 (1H, m, -CH-), 2.61 (2H, d, *J* = 8 Hz, -CH2-); ^13^C NMR (100 MHz, CDCl_3_, ppm): 188.45 (C-1), 120.21 (C-2), 141.67 (C-3), 128.63 (C-2′ and C-6′), 130.34 (C-3′ and C-5′), 134.22 (C-1′), 143.12 (C-4′), 134.71 (C-1′′), 144.51 (C-4′′), 128.12 (C-2′′ and C-6′′), 116.42 (C-3′′ and C-5′′), 21.96 (-CH_3_, C of isobutyl group at C-4′′), 28.29 (-CH-, C of isobutyl group at C-4′′), 45.34 (-CH_2_-, C of isobutyl group at C-4′′), 51.35 (-OCH_3_ C at C-4′′); MS (*m*/*z*, %): 311.1 (M + 1, 98.56); Anal. Calcd for: C_20_H_23_O_3_: C, 77.39; H, 7.14; Found: C, 77.44; H, 7.16.

#### 2.2.26. (E)-1-(4′-Isobutylphenyl)-3-(2′′-chloro-5′′-nitrophenyl)-2-propen-1-one (A25)

Yield 84%; m.p. 131–133°C; IR (KBr, cm^−1^): 1658 (C=O), 1603 (C=C of Ar), 1515 (CH=CH), 824 (C-Cl), 1525 (N=O, asymmetric), 1348 (N=O, symmetric), 3012 (Ar C-H), 2823 (Alkyl C-H); ^1^H NMR (400 MHz, CDCl_3_, ppm): *δ* 7.25 (1H, d, *J* = 17 Hz, -CO-CH=), 7.72 (1H, d, *J* = 17 Hz, =CH-Ar), 6.89–8.01 (7H, Ar-H), 1.02 (6H, d, *J* = 8 Hz, -(CH3)2), 2.25–2.41 (1H, m, -CH-), 2.65 (2H, d, *J* = 8 Hz, -CH2-); ^13^C NMR (100 MHz, CDCl_3_, ppm): *δ* 187.02 (C-1), 126.11 (C-2), 144.42 (C-3), 128.92 (C-2′ and C-6′), 130.24 (C-3′ and C-5′), 133.92 (C-1′), 147.13 (C-4′), 152.69 (C-2′′), 133.21 (C-3′′), 134.66 (C-4′′), 124.99 (C-5′′), 22.01 (-CH_3_, C of isobutyl group at C-4′′), 28.21 (-CH-, C of isobutyl group at C-4′′), 46.11 (-CH_2_-, C of isobutyl group at C-4′′); MS (*m*/*z*, %): 344.8 (M + 1, 99.0); Anal. Calcd for: C_19_H_18_ClNO_3_: C, 81.47; H, 7.22; N, 5.28; Found: C, 81.51; H, 7.25; N, 5.33.

### 2.3. Antimicrobial Evaluation

The antimicrobial activity was performed against four bacterial and two fungal strains. The organisms selected were the Gram-positive* Bacillus subtilis* (NCIM-2079,* Bs*),* Staphylococcus aureus *(NCIM-2063,* Sa*), Gram-negative* Escherichia coli *(NCIM-2068,* Ec*), and* Proteus vulgaris *(NCIM-2027,* Pv*) and the fungal strains* Aspergillus niger *(ATCC-6275,* An*) and* Candida tropicalis *(ATCC-1369,* Ca*). Serial tube dilution method was employed and the minimum inhibitory concentration was determined [40] for each compound. 2.048 mg of each test compound was placed in vials separately and 2 mL of methanol was added and a solution of the concentration 1.024 mg/mL was obtained. The test bacterium which was grown at 37°C in nutrient agar medium was diluted in sterile nutrient broth medium to get a suspension containing about 10^7^ cells/mL and was used as the inoculum. 11 test tubes were taken, 9 of which were marked as 1, 2, 3, 4, 5, 6, 7, 8, and 9 and the remaining two were assigned as T_M_ (medium) and T_MI_ (medium + inoculum). 1 mL of nutrient broth medium was poured into all the 11 test tubes, and they were cotton plugged and sterilized in an autoclave at 15 lbs/sq.in pressure. After cooling, 1 mL of the sample solution was added in the first test tube and mixed well and then 1 mL of this content was transferred to the second test tube and the process of serial dilution was continued up to the ninth test tube. 10 *μ*L of properly diluted inoculum was added to each of the nine test tubes and mixed well. 10 *μ*L of the inoculum was added to the test tube T_MI_ to observe the growth of the organism in the medium used. The controlled test tube T_M_ containing only the medium was used to confirm the sterility of the medium. All the test tubes were incubated at 37°C for 18 h. A similar experiment with medium, methanol, and inoculum without compound was also performed to ensure that the methanol has no inhibitory effect on the dilutions used. The test tube number in which the first sign of growth of the organism was observed was noted. The MIC was taken as that concentration used in the test tube number just prior to the test tube number where the first sign of growth was observed. This procedure was followed to determine the MIC values for all the compounds. The same procedure was followed for antifungal activity testing except that Potato-Dextrose-Agar medium was used.

### 2.4. Atom Based 3D-QSAR Studies of Chalcones

The employed methodology deals with development of atom based 3D-QSAR models to predict the antibacterial and antifungal activity for the synthesized chalcones. By these studies, it is possible to understand how the compounds interact with the target. The results emerging out of these studies can be used to identify new active ligands. For this reason, PHASE v 3.1 (Schrödinger LLC, Portland, Oregon, USA; https://www.schrodinger.com/) was used to carry out the defined studies.

#### 2.4.1. Data Set Selection

The data set consists of structurally diverse compounds having antibacterial and antifungal activities against* B. subtilis, S. aureus, E. coli, P. vulgaris, A. niger, *and* C. tropicalis*. The selected series of chalcones** (A1–A25) **and their antibacterial and antifungal activities are given in Table 1. The MIC (*µ*g/mL) values were taken into account to assess the antibacterial and antifungal activities of these compounds. The biological activities used in the present computational studies were represented as −log(MIC).

#### 2.4.2. Molecular Modeling (Energy Minimization)

The structures of all the 25 synthesized chalcones were modeled using Chemdraw Ultra 10.0 (Cambridge software), and then the modeled structure was copied to Chem3D Ultra 10.0 to create a 3D model which was subjected to energy minimization using molecular mechanics (MM2). The minimization was executed until the root-mean-square gradient value reached a value smaller than 0.001 kcal/mol. Such type of energy minimized structures was considered for molecular docking and 3D-QSAR studies. However, the corresponding MDL MOL files were prepared using Chem3D Ultra 10.0 integral options (save as /MDL MOL).

#### 2.4.3. Generation of 3D-QSAR Models Using PHASE

In order to understand the structural differences between the active and inactive compounds, it was thought that it was proper to do a visual inspection of the aligned ligands and quantify these differences by building a 3D-QSAR model that identifies which functional groups contribute, either positively or negatively, to activity. PHASE generated individual atom based 3D-QSAR models by using a set of 25 chalcones, which all have been aligned in a three-dimensional space with reference of their antibacterial and antifungal activity data. From this set of 25 compounds, 20 compounds were selected to generate the 3D-QSAR model (i.e., the training set; see supplementary data for tables (available here)) and 5 compounds were selected to validate it (i.e., the test set). The criterion used to select these sets was purely based on a random selection in the percent ratio of 80 : 20.

#### 2.4.4. PHASE'S Steps to Build the Atom Based 3D-QSAR Models

PHASE can use two alternative ways to generate the structural components that constitute the 3D-QSAR models, that is, the pharmacophore model and atom based 3D-QSAR. The atom based model is more useful for elucidating the structure activity relationships. In atom based 3D-QSAR models, a regression is performed by using the partial least-squares (PLS) method [where the independent variables are the binary-valued occupancies (i.e., the values are either 0 or 1) of the cubes by the different structural components]. Thus, in this model, the number of independent variables used is the 6*N* occupancies of the *N* cubes by the six available atom classes (i.e., each variable corresponds to a given cube and a given atom class) and the value for each variable can be 0 or 1. Therefore, the regression involves finding a linear least-squares relationship between the activity data (i.e., the dependent variable) and a special set of orthogonal factors that are linear combinations of the bit value variables (i.e., the independent variables). The accuracy of the 3D-QSAR models increases with increasing the number of PLS factors until overfitting starts to occur (where the maximum number of PLS factors is *N*/5 and *N* is the number of ligands). Thus, the PLS facilitates the identification of specific chemical features that tend to increase or decrease the estimated activity. The number of PLS used in the present study was 4. After that, the statistics for the training and test set were analyzed in order to produce a 3D-QSAR model with the best predictive power. Since the training sets employed in the present study have reduced structural diversity, the better model was obtained by using the atom based 3D-QSAR models. PHASE provides the means to build atom based 3D-QSAR models using the activities of the ligands without having a pharmacophore hypothesis in the Individual QSAR model panel.

#### 2.4.5. 3D-QSAR Validation and Statistics

PHASE supports only the use of a true external test set (i.e., compounds which have not been used to build the model) to validate the 3D- QSAR model. For this reason, it is necessary to analyze the statistics obtained from the training and test sets. The main statistical properties that describe the 3D-QSAR model when the training set data is used are as follows: (a) the *R*-squared or *R*^2^ (i.e., the coefficient of determination, which can never be negative); (b) the standard deviation of regression or SD; (c) the *F* statistic (i.e., the overall significance of the model); and (d) the statistical significance or *P* (i.e., the probability that the correlation could occur by chance). Thus, in the case that the independent variables have no statistical relationship with the activity, *R*^2^ would be 0. On the other hand, the main statistical quantities describing the test set prediction are as follows: (a) the *Q*-squared or *Q*^2^ (i.e., equivalent to *R*^2^ but now, using the predicted and experimental test set activity values, in contrast to *R*^2^, it can take negative values); (b) the Pearson value or *R* (i.e., the Pearson correlation coefficient); and (c) the root-mean-square error or RMSE. At this point, it is worth remarking that there is not any single parameter that allows choosing the best model. In this sense, we have to consider all the statistic parameters reported by PHASE to evaluate the different 3D-QSAR models.

#### 2.4.6. 3D-QSAR Model Visualization

**Figure 2:**
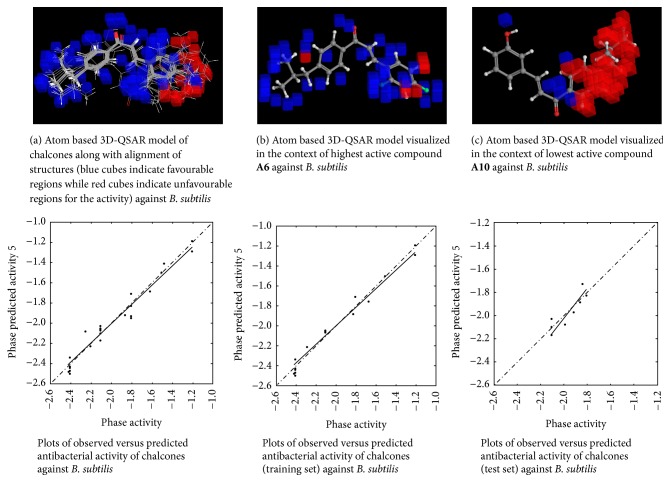
Atom based 3D-QSAR model for antibacterial activity of chalcones against* B. subtilis*.

**Figure 3:**
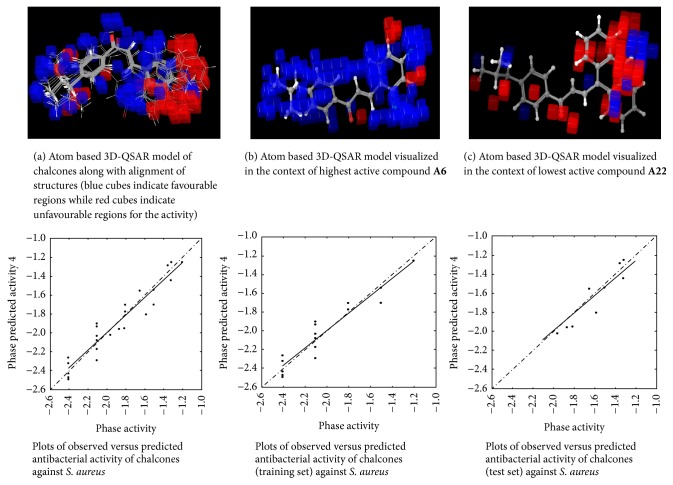
Atom based 3D-QSAR model for antibacterial activity of chalcones against* S. aureus*.

**Figure 4:**
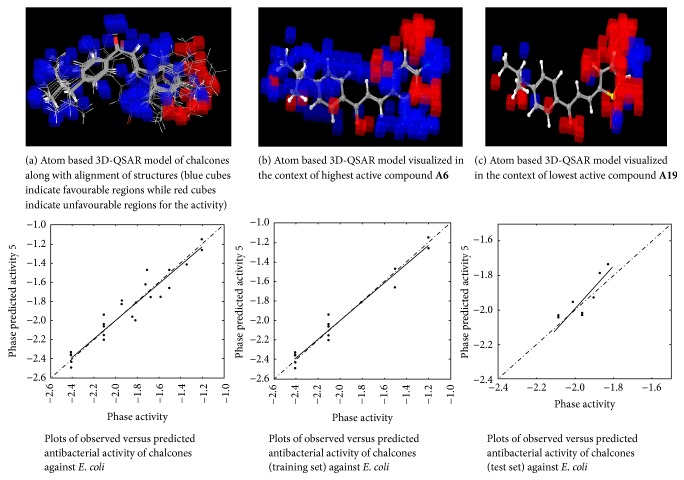
Atom based 3D-QSAR model for antibacterial activity of chalcones against* E. coli*.

**Figure 5:**
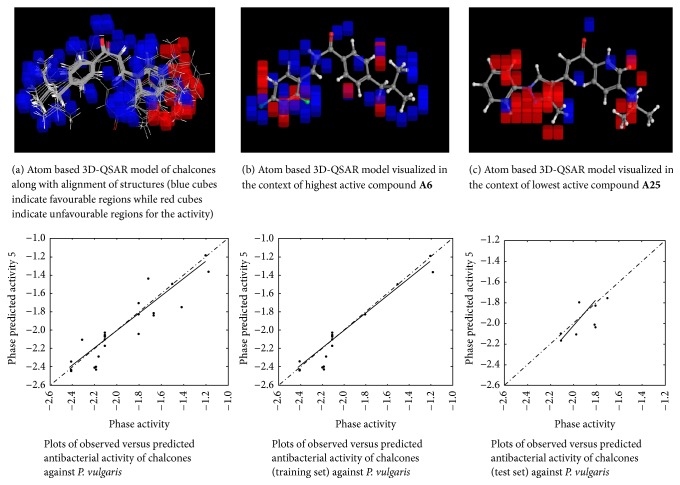
Atom based 3D-QSAR model for antibacterial activity of chalcones against* P. vulgaris*.

**Figure 6:**
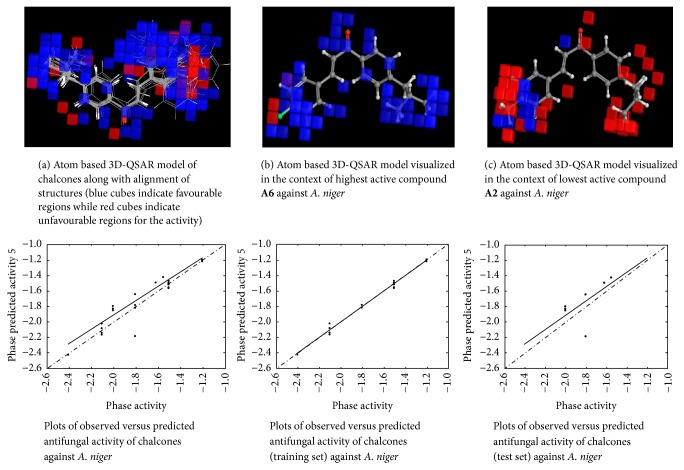
Atom based 3D-QSAR model for the antifungal activity of chalcones against* A. niger*.

**Figure 7:**
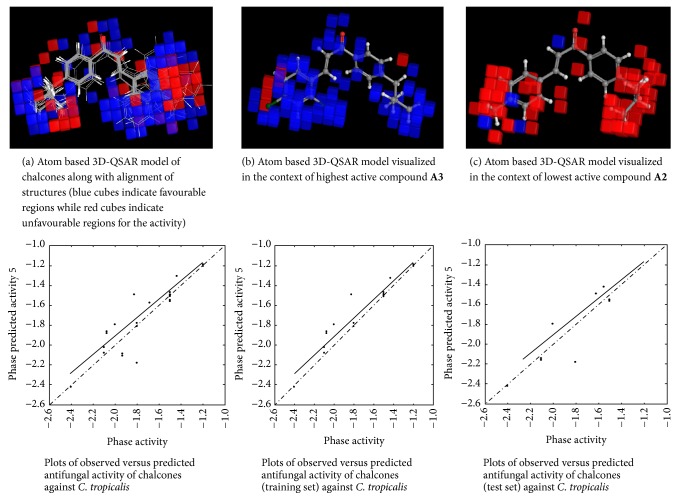
Atom based 3D-QSAR model for the antifungal activity of chalcones against* C. tropicalis*.

Once the 3D-QSAR models have been generated, we have to visualize and analyze them. Thus, to understand how the structures of the ligands contribute either positively or negatively to the computed activity, the three-dimensional aspects of the 3D-QSAR model were examined. The visualization allows viewing the cubic volume elements occupied by one specific ligand or all the cubes in the 3D-QSAR model which shows the volume occlusion maps (i.e., the union of the cubes occupied by all the compounds from the set). In this visualization, the maps represent the regions of favourable and unfavourable interactions. The volume occlusion maps describe the spatial arrangement of favourable interactions to accept groups of the target protein. In Figures 2–7 red region indicates unfavourable region for substitution and blue region indicates favourable region for substitution.

## 3. Results and Discussion

### 3.1. Chemistry

The conventional base-catalyzed Claisen-Schmidt condensation led to the synthesis of target compounds. Purification of the compounds was done by recrystallization employing ethanol as solvent. Structural elucidation of the compounds was done with the help of spectroscopic studies including IR, ^1^H NMR, ^13^C NMR, and MS. The spectral data of the compounds was in accordance with the anticipated structures. The IR spectra of these compounds give away characteristic -C=O stretching at 1645–1660 cm^−1^ and -C=C- stretching at 1450–1520 cm^−1^, respectively. Additional -C=C- and -C-H stretching at 1580–1610 cm^−1^ and 3010–3150 cm^−1^ had established the occurrence of aromatic rings. The ^1^H NMR spectra of the compounds were done by dissolving the compounds in CDCl_3_ and two diagnostic doublets in the spectrum around *δ* 6.7–7.4 and *δ* 7.3–7.8 ppm for -CO-CH= (*α*-H) and =CH-Ar (*β*-H), respectively, with coupling constant (*J* = 17 HZ) confirmed the formation and* trans* (*E*) arrangement of the chalcone bridge. Other protons exhibited additional resonance signals typically present in each compound. ^13^C NMR spectrum of the compounds publicized the characteristic signals around *δ* 186–191 (C-1), 120–128 (C-2), and 131–142 (C-3). The molecular ion peak in the mass spectrum further depicts the formation of chalcones. Elemental analysis results were within ±0.4% of the calculated values.

### 3.2. Antimicrobial Evaluation

The antimicrobial activity of the prepared chalcones was performed by serial tube method and their MIC values are summarized in Table 1. The standard drugs used were amoxicillin and fluconazole for antibacterial and antifungal activity, respectively, and the activity of all the tested compounds is less in comparison to the standard drugs (Table 1). The compounds** A3** and** A6** containing 2,4-dichlorophenyl and 2,4-difluorophenyl scaffolds at ring B portion of the chalcone bridge were found to be the most potent against all the bacterial and fungal strains with MIC value of 16 *µ*g/mL.** A1** having 4-chlorophenyl and** A5** containing 4-fluorophenyl substitution were next in potency with a MIC value of 32 *µ*g/mL against Gram-positive bacteria,* B. subtilis* and* S. aureus*, whereas** A25** with 2-chloro-5-nitrophenyl moiety exhibited potency with a MIC value of 32 *µ*g/mL against Gram-negative bacteria,* E. coli* and* P. vulgaris*. The compound** A5** with 4-fluorophenyl moiety is equipotent with** A3** and** A6** against* A. niger* and** A12** and** A17** with 4-methoxyphenyl and 4-pyridyl moieties exhibited similar potency as that of** A3** and** A6** against* C. tropicalis* with a MIC value of 16 *µ*g/mL. The other compounds carrying different electron withdrawing and electron releasing substituents were also found to be somewhat potent with MIC values ranging from 32 to 256 *µ*g/mL. A structure-activity-relationship study from the results indicated the necessity of electron withdrawing groups like chlorine, fluorine, and nitro groups at different positions on ring B. In addition a combination of electron withdrawing and releasing groups on the phenyl ring or heteroaryl rings can be synthesized and tested with a hope to get promising antimicrobial agents.

### 3.3. Atom Based 3D-QSAR Studies of Chalcones

The set of 25 chalcones** (A1–A25)** were subjected to atom based 3D-QSAR analysis using Partial Least Square (PLS) method to identify the potential antibacterial and antifungal agents. The statistical parameters for the activities are displayed in Table 2 and are valid. In the Figures 2–7 red region indicates unfavourable region for substitution, and blue region indicates favourable region for substitution. The orientation in these cubes gives a clue to find favourable and unfavourable functional groups for the biological activity.

The results clearly indicated the potential antibacterial activity of the chalcone** (A6)** having a 2,4-difluorophenyl moiety, as seen by more blue regions, and also proved the lowest activity of the chalcone** (A10)** as seen by fewer blue regions against* B. subtilis*. This computational observation was also consistent with the actual experimental results. These computational studies could predict the lowest antibacterial activity of the chalcone** (A10)** with 3-hydroxyphenyl moiety and this observation again was consistent with the actual results. Moreover this indicates the superior predictive ability of computational models. The chalcone** (A6)** having a 2,4-difluorophenyl moiety is capable of showing significant antibacterial activity and compound** (A22)** having 9-anthracenyl showed the least activity as evidenced by these computational studies against* S. aureus*. This was again consistent with the actual results. However, in the actual results some other compounds could also show similar effect against* S. aureus*. The chalcone** (A6)** also is capable of showing significant antibacterial activity and** (A19)** showed the least activity against* E. coli*. The chalcone** (A6)** having a 2,4-difluorophenyl substitution is capable of showing maximum antibacterial activity and** A23** showed the least activity against* P. vulgaris* as evidenced by these computational studies, which was again consistent with the actual results. The compound** A6** emerged as the most promising molecule while the compound** A2** became the least potent compound against* A. niger*. Similarly the compound** A3** was identified as the most promising and** A2** as the least potent compound against* C. tropicalis*.

## 4. Conclusion

We report the synthesis, structural elucidation, and antimicrobial activities of a series of 4-isobutylacetophenone chalcones. These compounds can be synthesized in good yields. The compounds** A3** and** A6** exhibited prominent antibacterial and antifungal activity. In each case the contribution of electron withdrawing groups is remarkable in enhancing the activity. Further optimization on the leads can be attempted by introducing substituents on the 4-isobutylacetophenone moiety or on ring B portion in order to increase the activity. The observed −log(MIC) values from the actual results and the predicted −log(MIC) values through PLS method were well correlated, supporting the usefulness of these computational studies.

## Figures and Tables

**Table 1:** Antibacterial and antifungal activities of chalcones (MIC in *µ*g/mL).

Compound	**R**	*Bs*	*Sa*	*Ec*	*Pv*	*An*	*Ct*
**A1**	4′′-Chlorophenyl	32	32	64	64	32	64
**A2**	4′′-Methylphenyl	256	128	128	128	256	256
**A3**	*2*′′*,4*′′*-Dichlorophenyl*	*16*	*16*	*16*	*16*	*16*	*16*
**A4**	2′′-Chlorophenyl	64	64	128	128	64	64
**A5**	4′′-Fluorophenyl	32	32	64	64	16	32
**A6**	*2*′′*,4*′′*-Difluorophenyl*	*16*	*16*	*16*	*16*	*16*	*16*
**A7**	4′′-Dimethylaminophenyl	256	128	256	256	128	64
**A8**	3′′-Bromophenyl	128	128	128	128	64	128
**A9**	4′′-Hydroxyphenyl	64	64	64	64	128	128
**A10**	3′′-Hydroxyphenyl	256	256	128	256	128	64
**A11**	4′′-Nitrophenyl	128	64	64	128	64	64
**A12**	4′′-Methoxyphenyl	128	128	256	128	32	*16*
**A13**	3′′,4′′-Dimethoxyphenyl	256	128	256	128	64	128
**A14**	3′′,4′′,5′′-Trimethoxyphenyl	128	128	128	256	32	32
**A15**	2′′-Pyridinyl	64	128	64	128	32	64
**A16**	3′′-Pyridinyl	64	128	128	64	64	128
**A17**	4′′-Pyridinyl	64	128	128	64	32	*16*
**A18**	2′′-Pyrrolyl	128	256	128	256	64	32
**A19**	2′′-Thienyl	128	256	256	256	32	64
**A20**	5′′-Bromofuran-2′′-yl	64	128	64	128	64	32
**A21**	3′′,4′′-Methylenedioxyphenyl	256	256	64	64	64	128
**A22**	9′′-Anthracenyl	128	256	128	128	32	32
**A23**	1′′-Phenyl-3′′-methylpyrazole-4′′-yl	256	128	128	256	128	64
**A24**	3′′-Methoxy-4′′-hydroxyphenyl	264	128	64	64	64	32
**A25**	2′′-Chloro-5-nitrophenyl	64	64	32	32	64	32
**Ampicillin**		<1	<1	<1	<1	--	--
**Fluconazole**		--	--	--	--	<2	<2

*Bs = Bacillus subtilis; Sa = Staphylococcus aureus; Ec = Escherichia coli; Pv = Proteus vulgaris; An = Aspergillus niger; Ct = Candida tropicalis*.

**Table 2:** Summary of the atom based 3D-QSAR statistical data results.

Organism	PLS factors	SD	*R* ^2^	*F*	*P*	RMSE	*Q*-squared	Pearson-*R*
*B. subtilis*	5	0.1405	0.9296	17.9	5.19*e* − 06	0.22	0.5219	0.6622
*S. aureus*	4	0.1345	0.9211	35	1.574*e* − 06	0.14	0.4623	0.7484
*E. coli*	5	0.0963	0.9623	56.2	1.848*e* − 07	0.15	0.4529	0.7285
*P. vulgaris*	5	0.1081	0.7262	51.5	1.507*e* − 07	0.28	0.5607	0.788
*A. niger*	5	0.041	0.9215	56.1	1.232*e* − 07	0.327	0.4529	0.7285
*C. tropicalis*	5	0.1068	0.9312	38.2	1.638*e* − 06	0.27	0.566	0.7109

## Supplementary Materials

Supplementary Materials Table 3: experimental and predicted MIC (*μ*g/mL) values of training set and test set molecules based on atom based 3D-QSAR model (antibacterial activity). Table 4: experimental and predicted MIC (*μ*g/mL) values of training set and test set molecules based on atom based 3D-QSAR model (antibacterial activity). Table 5: experimental and predicted MIC (*μ*g/mL) values of training set and test set molecules based on atom based 3D-QSAR model (antibacterial activity). Table 6: experimental and predicted MIC (*μ*g/mL) values of training set and test set molecules based on atom based 3D-QSAR model (antibacterial activity). Table 7: experimental and predicted MIC (*μ*g/mL) values of training set and test set molecules based on atom based 3D-QSAR model (antifungal activity). Table 8: experimental and predicted MIC (*μ*g/mL) values of training set and test set molecules based on atom based 3D-QSAR model (antifungal activity).

## References

[1] Bax R., Mullan N., Verhoef J. (2000). The millennium bugs—the need for and development of new antibacterials. *International Journal of Antimicrobial Agents*.

[2] Coates A., Hu Y., Bax R., Page C. (2002). The future challenges facing the development of new antimicrobial drugs. *Nature Reviews Drug Discovery*.

[3] Barrett C. T., Barrett J. F. (2003). Antibacterials: Are the new entries enough to deal with the emerging resistance problems?. *Current Opinion in Biotechnology*.

[4] Tunçbilek M., Kiper T., Altanlar N. (2009). Synthesis and in vitro antimicrobial activity of some novel substituted benzimidazole derivatives having potent activity against MRSA. *European Journal of Medicinal Chemistry*.

[5] Burger (2003). *Burger’s Medicinal Chemistry and Drug Discovery*.

[6] Zimmerman B. E., Zimmerman D. J. (2003). *Microbes and Diseases That Threaten Humanity*.

[7] Bryskier A., Bryskier A. (2005). In pursuit of new antibiotics. *Antimicrobial Agents*.

[8] Shaik Y., Vidya S. G., Shaik A. B. (2015). Biological and synthetic potentiality of chalcones: a review. *Journal of Chemical and Pharmaceutical Research*.

[9] Batovska D. I., Todorova I. T. (2010). Trends in utilization of the pharmacological potential of chalcones. *Current Clinical Pharmacology*.

[10] Sahu N. K., Balbhadra S. S., Choudhary J., Kohli D. V. (2012). Exploring pharmacological significance of chalcone scaffold: a review. *Current Medicinal Chemistry*.

[11] Dimmock J. R., Elias D. W., Beazely M. A., Kandepu N. M. (1999). Bioactivities of chalcones. *Current Medicinal Chemistry*.

[12] Gaikwad S. S., Suryawanshi V. S., Lohar K. S., Jadhav D. V., Shinde N. D. (2012). Synthesis and biological activity of some 3,4-dihydro-4-(4-substituted aryl)-6-(naptho[2,1-b]furan-2-yl pyrimidine-2(1H)-one derivatives. *E-Journal of Chemistry*.

[13] Suwito H., Ni'matuzahroh, Kristanti A. N. (2016). Antimicrobial activities and in silico analysis of methoxy amino chalcone derivatives. *Procedia Chemistry*.

[14] Solankee A., Patel K., Patel R. (2012). A facile synthesis and studies of some new chalcones and their derivatives based on heterocyclic ring. *E-Journal of Chemistry*.

[15] Liaras K., Geronikaki A., Glamočlija J., Ćirić A., Soković M. (2011). Thiazole-based chalcones as potent antimicrobial agents. Synthesis and biological evaluation. *Bioorganic & Medicinal Chemistry*.

[16] Sudhir P., Rajashree C., Ashok B. (2012). Synthesis and biological evaluation of mannich bases of isoxazoline derivatives as novel anti-microbial agents. *E-Journal of Chemistry*.

[17] Sharma V., Singh G., Kaur H., Saxena A. K., Ishar M. P. S. (2012). Synthesis of *β*-ionone derived chalcones as potent antimicrobial agents. *Bioorganic & Medicinal Chemistry Letters*.

[18] Ahmad A., Husain A., Khan S. A., Mujeeb M., Bhandari A. (2016). Synthesis, antimicrobial and antitubercular activities of some novel pyrazoline derivatives. *Journal of Saudi Chemical Society*.

[19] Ahmad I., Thakur J. P., Chanda D. (2013). Syntheses of lipophilic chalcones and their conformationally restricted analogues as antitubercular agents. *Bioorganic & Medicinal Chemistry Letters*.

[20] Coskun D., Erkisa M., Ulukaya E., Coskun M. F., Ari F. (2017). Novel 1-(7-ethoxy-1-benzofuran-2-yl) substituted chalcone derivatives: synthesis, characterization and anticancer activity. *European Journal of Medicinal Chemistry*.

[21] Zhang J., Fu X.-L., Yang N., Wang Q.-A. (2013). Synthesis and cytotoxicity of chalcones and 5-deoxyflavonoids. *The Scientific World Journal*.

[22] Zhang J., Yang F., Qiao Z., Zhu M., Zhou H. (2016). Chalcone–benzoxaborole hybrids as novel anticancer agents. *Bioorganic & Medicinal Chemistry Letters*.

[23] Coşkun D., Tekin S., Sandal S., Coşkun M. F. (2016). Synthesis, characterization, and anticancer activity of new benzofuran substituted chalcones. *Journal of Chemistry*.

[24] Sridevi C., Balaji K., Naidu A. (2011). Synthesis and pharmacological evaluation of some phenylpyrazolo indoquinoxaline derivatives. *E-Journal of Chemistry*.

[25] Venkatachalam H., Nayak Y., Jayashree B. S. (2012). Synthesis, characterization and antioxidant activities of synthetic chalcones and flavones. *APCBEE Procedia*.

[26] Hayat F., Moseley E., Salahuddin A., van Zyl R. L., Azam A. (2011). Antiprotozoal activity of chloroquinoline based chalcones. *European Journal of Medicinal Chemistry*.

[27] Lakshmi K., Rao N. R., Basaveswararao M. V. (2014). Synthesis, antimicrobial and anthelmintic evaluation of novel quinazolinonyl chalcones. *Rasayan Journal of Chemistry*.

[28] Pingaew R., Saekee A., Mandi P. (2014). Synthesis, biological evaluation and molecular docking of novel chalcone-coumarin hybrids as anticancer and antimalarial agents. *European Journal of Medicinal Chemistry*.

[29] Choudhary A. N., Kumar A., Juyal V. (2012). Design, synthesis and evaluation of chalcone derivatives as anti-inflammatory, antioxidant and antiulcer agents. *Letters in Drug Design and Discovery*.

[30] Sridhar S., Dinda S. C., Prasad Y. R. (2011). Synthesis and biological evaluation of some new chalcones containing 2,5-dimethylfuran moiety. *E-Journal of Chemistry*.

[31] Sridhar S., Rajendraprasad Y. (2012). Synthesis and analgesic studies of some new 2-pyrazolines. *E-Journal of Chemistry*.

[32] Rani P., Srivastava V. K., Kumar A. (2004). Synthesis and antiinflammatory activity of heterocyclic indole derivatives. *European Journal of Medicinal Chemistry*.

[33] Das M., Manna K. (2016). Chalcone scaffold in anticancer armamentarium: a molecular insight. *Journal of Toxicology*.

[34] Nowakowska Z. (2007). A review of anti-infective and anti-inflammatory chalcones. *European Journal of Medicinal Chemistry*.

[35] Suwito H., Ni'matuzahroh, Kristanti A. N. (2016). Antimicrobial activities and in silico analysis of methoxy amino chalcone derivatives. *Procedia Chemistry*.

[36] Vanangamudi G., Subramanian M., Thirunarayanan G. (2017). Synthesis, spectral linearity, antimicrobial, antioxidant and insect antifeedant activities of some 2,5-dimethyl-3-thienyl chalcones. *Arabian Journal of Chemistry*.

[37] Shaik A. B., Prasad Y. R., Shahanaaz S. (2015). Design, facile synthesis, characterization and computational evaluation of novel isobutylchalcones as cytotoxic agents: part-A. *Fabad Journal of Pharmaceutical Sciences*.

[38] Srinath N., Prasasd Y. R., Mukkanti K., Kistayya C., Rao B. B. (2011). Synthesis and anti-inflammatory activity of some new chalcones from 3'-methyl-4'-hydroxyacetophenone. *Current Trends in Biotechnology and Pharmacy*.

[39] Ravindar B., Srinivasa M. M., Shaik A. B. (2016). Design, facile synthesis, and biological evaluation of novel 1,3-thiazine derivatives as potential anticonvulsant agents. *Asian Journal of Pharmaceutical and Clinical Research*.

[40] Andrews J. M. (2001). Determination of minimum inhibitory concentrations. *Journal of Antimicrobial Chemotherapy*.

