# Supplementary material for: Synthesis, Antimicrobial, and Computational Evaluation of Novel Isobutylchalcones as Antimicrobial Agents
Source: Int J Med Chem. 2017 Dec 26;2017:6873924. doi: 10.1155/2017/6873924 (PMC5758854; doi:10.1155/2017/6873924)
Supplement: Supplementary Materials — Table 3: experimental and predicted MIC (μg/mL) values of training set and test set molecules based on atom based 3D-QSAR model (antibacterial activity). Table 4: experimental and predicted MIC (μg/mL) values of training set and test set molecules based on atom based 3D-QSAR model (antibacterial activity). Table 5: experimental and predicted MIC (μg/mL) values of training set and test set molecules based on atom based 3D-QSAR model (antibacterial activity). Table 6: experimental and predicted MIC (μg/mL) values of training set and test set molecules based on atom based 3D-QSAR model (antibacterial activity). Table 7: experimental and predicted MIC (μg/mL) values of training set and test set molecules based on atom based 3D-QSAR model (antifungal activity). Table 8: experimental and predicted MIC (μg/mL) values of training set and test set molecules based on atom based 3D-QSAR model (antifungal activity). [file 6873924.f1.pdf]

Table 3. Experimental and predicted MIC (μg/mL) values of training set and test set molecules based on atom based 3D-QSAR model (Antibacterial activity).

Compound code	*B.subtilis* MIC (μg/mL)	Experimental -log(MIC)	Predicted -log(MIC) (Training set)	Predicted -log(MIC) (Test set)
**A1**	32	-1.505	-1.74	
**A2**	256	-2.408	-2.11	
**A3**	16	-1.204	-1.19	
**A4**	64	-1.806		-1.31
**A5**	32	-1.505	-1.50	
**A6**	16	-1.204	-1.29	
**A7**	256	-2.408	-2.44	
**A8**	128	-2.107		-2.17
**A9**	64	-1.806	-1.71	
**A10**	256	-2.408	-2.34	
**A11**	128	-2.107	-2.07	
**A12**	128	-2.107	-2.10	
**A13**	256	-2.408	-2.47	
**A14**	128	-2.107		-2.35
**A15**	64	-1.806		-1.83
**A16**	64	-1.806		-1.93
**A17**	64	-1.806	-1.71	
**A18**	128	-2.107	-2.05	
**A19**	128	-2.107	-2.06	
**A20**	64	-1.806		-2.04
**A21**	256	-2.408	-2.50	
**A22**	128	-2.107		-2.03
**A23**	256	-2.408	-2.43	
**A24**	264	-2.422	-2.48	
**A25**	64	-1.806	-1.95	

Table 4. Experimental and predicted MIC (μg/mL) values of training set and test set molecules based on atom based 3D-QSAR model (Antibacterial activity).

Compound code	*S. aureus* MIC (μg/mL)	Experimental -log(MIC)	Predicted -log(MIC) (Training set)	Predicted -log(MIC) (Test set)
**A1**	32	-1.50515	-1.55	
**A2**	128	-2.10721	-1.94	
**A3**	16	-1.20412	-1.26	
**A4**	64	-1.80618	-1.78	
**A5**	32	-1.50515	-1.71	
**A6**	16	-1.20412	-1.26	
**A7**	128	-2.10721		-2.2
**A8**	128	-2.10721	-1.91	
**A9**	64	-1.80618		-1.95
**A10**	256	-2.40824	-2.27	
**A11**	64	-1.80618	-1.71	
**A12**	128	-2.10721		-2.02
**A13**	128	-2.10721		-2.28
**A14**	128	-2.10721	-2.08	
**A15**	128	-2.10721	-2.18	
**A16**	128	-2.10721		-1.95
**A17**	128	-2.10721	-2.04	
**A18**	256	-2.40824	-2.5	
**A19**	256	-2.40824	-2.48	
**A20**	128	-2.10721		-2.05
**A21**	256	-2.40824	-2.43	
**A22**	256	-2.40824	-2.33	
**A23**	128	-2.10721		-2.25
**A24**	128	-2.10721	-2.3	
**A25**	64	-1.80618		-1.55

Table 5. Experimental and predicted MIC (μg/mL) values of training set and test set molecules based on atom based 3D-QSAR model (Antibacterial activity).

Compound code	*E. coli* MIC (μg/mL)	Experimental -log(MIC)	Predicted -log(MIC) (Training set)	Predicted -log(MIC) (Test set)
**A1**	64	-1.806	-1.82	
**A2**	128	-2.107	-2.37	
**A3**	16	-1.204		-1.42
**A4**	128	-2.107	-1.99	
**A5**	64	-1.806	-1.76	
**A6**	16	-1.204	-1.50	
**A7**	256	-2.408		-2.64
**A8**	128	-2.107		-2.32
**A9**	64	-1.806	-1.75	
**A10**	128	-2.107	-2.34	
**A11**	64	-1.806		-1.78
**A12**	256	-2.408	-2.26	
**A13**	256	-2.408	-2.35	
**A14**	128	-2.107	-2.31	
**A15**	64	-1.806		-1.71
**A16**	128	-2.107	-2.12	
**A17**	128	-2.107	-1.86	
**A18**	128	-2.107		-2.46
**A19**	256	-2.408	-2.28	
**A20**	64	-1.806	-1.75	
**A21**	64	-1.806	-1.79	
**A22**	128	-2.107		-2.32
**A23**	128	-2.107		-2.29
**A24**	64	-1.806	-1.92	
**A25**	32	-1.505	-1.43	

Table 6. Experimental and predicted MIC (μg/mL) values of training set and test set molecules based on atom based 3D-QSAR model (Antibacterial activity).

Compound code	*P. vulgaris* MIC (μg/mL)	Experimental -log(MIC)	Predicted -log(MIC) (Training set)	Predicted -log(MIC) (Test set)
**A1**	64	-1.806		-1.826
**A2**	128	-2.107		-2.35
**A3**	16	-1.204	-1.48	
**A4**	128	-2.107	-1.95	
**A5**	64	-1.806	-1.78	
**A6**	16	-1.204	-1.52	
**A7**	256	-2.408	-2.22	
**A8**	128	-2.107		-2.21
**A9**	64	-1.806	-1.75	
**A10**	256	-2.408	-2.48	
**A11**	128	-2.107	-2.31	
**A12**	128	-2.107		-2.31
**A13**	128	-2.107		-2.39
**A14**	256	-2.408	-2.38	
**A15**	128	-2.107	-2.21	
**A16**	64	-1.806	-1.75	
**A17**	64	-1.806	-1.89	
**A18**	256	-2.408	-2.46	
**A19**	256	-2.408	-2.26	
**A20**	128	-2.107	-2.15	
**A21**	64	-1.806		-1.86
**A22**	128	-2.107	-2.18	
**A23**	256	-2.408		-2.28
**A24**	64	-1.806		-1.69
**A25**	32	-1.505	-1.68	

Table 7. Experimental and predicted MIC (μg/mL) values of training set and test set molecules based on atom based 3D-QSAR model (Antifungal activity).

Compound code	*A. niger* MIC (μg/mL)	Experimental -log(MIC)	Predicted -log(MIC) (Training set)	Predicted -log(MIC) (Test set)
A1	32	-1.505	-1.51	
A2	256	-2.408	-2.42	
A3	16	-1.204	-1.21	
A4	64	-1.806		-1.44
A5	16	-1.204	-1.21	
A6	16	-1.204	-1.19	
A7	128	-2.107	-2.08	
A8	64	-1.806		-1.64
A9	128	-2.107	-2.02	
A10	128	-2.107	-2.16	
A11	64	-1.806	-1.81	
A12	32	-1.505	-1.50	
A13	64	-1.806		-1.35
A14	32	-1.505	-1.55	
A15	32	-1.505	-1.47	
A16	64	-1.806		-1.51
A17	32	-1.505	-1.56	
A18	64	-1.806		-1.44
A19	32	-1.505	-1.49	
A20	64	-1.806		-1.54
A21	64	-1.806		-2.18
A22	32	-1.505	-1.51	
A23	128	-2.107	-2.14	
A24	64	-1.806	-1.78	
A25	64	-1.806		-1.56

Table 8. Experimental and predicted MIC (μg/mL) values of training set and test set molecules based on atom based 3D-QSAR model (Antifungal activity).

Compound code	*C. tropicalis* MIC (μg/mL)	Experimental -log(MIC)	Predicted -log(MIC) (Training set)	Predicted -log(MIC) (Test set)
**A1**	64	-1.806	-1.959	
**A2**	256	-2.408	-2.52	
**A3**	16	-1.204		-1.31
**A4**	64	-1.806		-1.99
**A5**	32	-1.505	-1.47	
**A6**	16	-1.204	-1.31	
**A7**	64	-1.806	-1.99	
**A8**	128	-2.107		-2.32
**A9**	128	-2.107	-2.35	
**A10**	64	-1.806		-1.68
**A11**	64	-1.806	-2.01	
**A12**	16	-1.204	-1.46	
**A13**	128	-2.107	-2.36	
**A14**	32	-1.505	-1.45	
**A15**	64	-1.806	-2.04	
**A16**	128	-2.107	-2.25	
**A17**	16	-1.204	-1.42	
**A18**	32	-1.505		-1.59
**A19**	64	-1.806	-1.93	
**A20**	32	-1.505	-1.64	
**A21**	128	-2.107		-2.34
**A22**	32	-1.505	-1.45	
**A23**	64	-1.806		-1.98
**A24**	32	-1.505	-1.45	
**A25**	32	-1.505		-1.72
